# Glances at the Hospitals

**Published:** 1904-05-28

**Authors:** 


### GLANCES AT THE HOSPITALS.

### WEST SUFFOLK HOSPITAL.

The income for the year fell short of the expenditure by £611, and to meet this deficit a sum of £400 was paid from the reserve fund, and £111 has been carried forward to the next account, the remaining £100 being apparently derived from Lord John Hervey's legacy. A new operating theatre has been finished at a cost of £3,200. There were 49 in-patients remaining from the previous year, and 534 were admitted subsequently. Of the total, 343 were cured, 58 relieved, 30 died, 106 left for various reasons, and 46 were under treatment on the date of the report. The average stay in the hospital was 38 days—a rather long period. Merely a synopsis of the diseases treated is given. There are no medical or surgical tables in the ordinary meaning of the word, and although 247 operations were performed, there is no table of the anæsthetics used.

### GLASGOW FEVER AND SMALL-POX HOSPITALS, BELVIDERE.

At the beginning of the year 91 patients were in residence and 2,977 were admitted during it. Of these 2,458 were discharged cured or relieved, and 300 died. The daily average number resident was 319; the average stay in the hospital was nearly 36 days, and the mortality was 10 1 per cent. The enteric fever cases reached a total of 338, with 67 deaths, the mortality being 19·9 per cent. Many of these enteric cases were complicated with other diseases. For instance 5 had pneumonia, 4 had bronchitis, and 1 had pleurisy. Thirty-four had hæmorrhage and 10 had perforation. There were 19 cases of typhus fever with 5 deaths, showing a mortality of 26 3 per cent. Diphtheria was represented by 142 cases. Of this total 77 are classified as "faucial cases." Thirty-six of these were males, and gave a mortality of 4 or 11·1 per cent., 41 were females with a mortality of 8 or 19·5 per cent. Sixty-five were "laryngeal cases," and of 30 males 8 died, being a mortality of 26·6 per cent.; of 35 females 8 died, being a mortality of 22·9 per cent. There were 33 tracheotomies with 10 deaths; the males showing a mortality after this operation of nearly 50 per cent., and the females a little over 15 per cent. Of 865 cases of scarlatina, there were 29 deaths, being a mortality of 3·3 per cent. Ten cases of plague were admitted and 2 of them died. Not fewer than 73 patients were admitted with non-infectious diseases, and 27 were admitted without any disease being discovered. Of course pneumonia will occasionally be mistaken for typhus or other fever, but we find the high number of 30 cases being sent in to the Glasgow Fever Hospital in one year, and in addition to this number broncho-pneumonia figures 8 times and tonsillitis 9 times. Table XI. is a very instructive one for it shows that 4 patients were admitted who had both measles and scarlatina, 2 who had measles and chicken-pox, 8 who had measles and whooping-cough, 3 who had scarlatina and whooping-cough, and 1 with scarlatina and chicken-pox. At the small-pox hospital 604 cases were admitted. Of these 488 were small-pox, 75 chicken-pox alone or with other diseases, and, in addition to these, 10 different diseases were present, eczema, pemphigus, syphilis, and even nephritis being represented. Of the 488 small-pox cases, 37 were unvaccinated, of whom 25 recovered and 12 died, giving a mortality of 32·4 per cent. Of the 437 vaccinated cases, 26 died, giving a mortality of 5·99 per cent. There were 17 cases in which vaccination was doubtful, and of these 35·3 per cent. died. The tables are all drawn out with much care and precision.

